# Knockdown of long non-coding RNA NEAT1 inhibits glioma cell migration and invasion via modulation of *SOX2* targeted by miR-132

**DOI:** 10.1186/s12943-018-0849-2

**Published:** 2018-07-27

**Authors:** Ke Zhou, Chi Zhang, Hui Yao, Xuewen Zhang, Youxin Zhou, Yanjun Che, Yulun Huang

**Affiliations:** 1 Department of Neurosurgery, the First Affiliated Hospital of Soochow University, No.188, Shizi Street, Suzhou, 215007 Jiangsu China; 2 Department of Neurosurgery, Jingjiang People’s Hospital, No. 28 East Zhongzhou Road, Taizhou, 214500 Jiangsu China

**Keywords:** Glioma, microRNA-132, NEAT1, *SOX2*, Invasion, Migration

## Abstract

**Background:**

A better understanding of the molecular mechanism involving lncRNA-miRNA-mRNA network underlying glioma genesis is beneficial to the treatment of glioma. This study was designed to investigate the role of lncRNA NEAT1, miR-132 and *SOX2* interaction in glioma.

**Methods:**

Microarray analysis was conducted to identify the differentially expressed lncRNAs in glioma tissues. The expression levels of NEAT1, miR-132 and *SOX2* were determined by qRT-PCR and western blot. Proliferation of glioma cells was detected by MTT assay, while migration and invasion were determined by transwell assay. The target relationships were predicted by miRcode algorithm, and confirmed by dual luciferase reporter gene assay.

**Results:**

NEAT1 was up-regulated in glioma. Knockdown of NEAT1 inhibited glioma cells’ viability, migration and invasion. MiR-132 was down-regulated while *SOX2* was up-regulated in glioma cells. NEAT1 negatively regulated the expression of miR-132 in glioma while miR-132 targeted *SOX2* to down-regulate its expression.

**Conclusion:**

NEAT1 promoted glioma development by promoting *SOX2* expression through suppressing miR-132.

**Electronic supplementary material:**

The online version of this article (10.1186/s12943-018-0849-2) contains supplementary material, which is available to authorized users.

## Background

As the most common and aggressive primary brain tumor, glioma is characterized with extremely poor prognosis outcomes [1]. The overall survival of low-grade glioma patients is approximately 60 months, and that for high-grade glioma is only about 13 months [2]. Invasive growth happens more frequently in glioma patients, which makes complete tumor resection very difficult, leading to a high rate of lethality [3]. Despite of the considerable progresses achieved in neuro-oncology, the mortality of glioma still remains high [4]. A previous study indicated that abnormal expression of long non-coding RNAs (lncRNAs) and microRNAs (miRNAs), as well as their interaction in gliomas, could be new molecular targets for glioma therapy [3]. Thus, a better understanding of molecular mechanisms underlying glioma genesis is beneficial to the treatment of glioma.

LncRNAs are a prominent class of RNAs sized 200+ nucleotides, which could control gene expression at transcriptional and posttranscriptional levels, inactivating or stabilizing proteins [5]. Lots of studies have shown that the dysregulation of lncRNAs may influence cell proliferation and apoptosis, and engage in tumorigenesis and drug resistance. LncRNA nuclear paraspeckle assembly transcript 1 (NEAT1) was reported as an oncogenic lncRNA in several types of human cancers. For instance, its upregulation has been documented in kidney cancer, ovarian cancer, lung cancer, breast cancer and glioma [6]. NEAT1 could facilitate the initiation of hepatocellular carcinoma and predict poor prognosis outcome [7]. NEAT1 knockdown in glioma stem cells could restrain cell invasiveness and promote cell apoptosis resulted from the activation of let-7e [8]. In addition, Chen et al. suggested that through affecting the WNT/β-Catenin pathway, NEAT1 contributed to the worsening of glioblastoma [9]. In brief, lncRNA NEAT1 was suggested as a tumor promotor in many malignancies, including glioma.

On the other hand, miRNAs are highly conserved small single-stranded non-coding RNAs, which could directly regulate gene expression through inhibiting the translation or promoting the degradation of mRNA [10]. Mounting evidence suggested that the dysregulation of miRNAs contributes to the development of cancers, such as miR-132. Chen et al. reported the inhibitory effects of miR-132 on the proliferation, metastasis and radio-resistance of glioma cells [11]. Li et al. also demonstrated that miR-132 inhibited the tumorigenicity of glioma cells and promoted their apoptosis [12]. The suppressive influence that miR-132 imposed on glioma deserves further studies.

Sex determining region Y-box protein 2 (*SOX2*), a transcription factor involves in pluripotency induction and maintenance of cell stemness, has been proved to be aberrantly expressed in various solid tumors including breast cancer, lung cancer, prostate cancer, glioblastomas and melanomas [13]. *SOX2* is important for the survival of glioma stem cells and closely associated with the relapse of glioma after chemotherapy or radio-therapy in adults [14]. Dong et al. suggested that *SOX2* was an oncogene in glioblastoma multiforme and *SOX2* down-regulation could suppress the proliferation, invasion and migration of cancer cells [15]. Although NEAT1, miR-132 and *SOX2* have been proved to play crucial roles in glioma, their regulatory mechanism and interaction still need to be further studied.

Therefore, we investigated the interaction between lncRNA NEAT1, miR-132 and *SOX2* in glioma and identified their roles in glioma. NEAT1 could indirectly regulate *SOX2* expression through targeting miR-132. NEAT1 and *SOX2* knockdown successfully reduced the invasiveness of glioma cells. These findings can provide new insights into glioma treatment.

## Methods

### Tissue samples

Resected brain tumors were collected from Jingjiang People’s Hospital from Jan 2012 to Jan 2016. Tissue samples included 14 glioma samples and 5 adjacent non-tumor samples. Tumor samples were pathologically graded as 7 low grade tumors (stage I and stage II) and 7 high grade tumors (stage III and stage IV) according to the WHO criteria. All tissues were directly preserved in liquid nitrogen and stored at − 80 °C. In this study, all investigation and experiments have obtained patients’ consent and been approved by the Ethic Committee for Clinical Research of Jingjiang People’s Hospital. The baseline characteristics of the included patients are shown in Additional file 1: Table S1.

### Microarray profiling

Total RNA was extracted from 14 fresh human glioma tissues and five normal tissues using TRIzol Reagent (Invitrogen, Carlsbad, CA) and purified with a RNeasy Mini Kit (Qiagen, Valencia, CA). Besides, sample preparation and microarray hybridization were performed based on the manufacturer’s standard protocols with minor modifications. LncRNAs with differential expressions in glioma tissues were picked out by the whole genome microarray expression profiling with the criteria of log_2_ (fold change) > 2 and adjusted *P* < 0.01. Sample labeling, microarray hybridization and washing were all performed according to the manufacturer’s standard protocols. Briefly, Cyanine-3-CTPlabeled cRNA was hybridized onto the lncRNA microarray chips (Affymetrix Human Genome U133 Plus 2.0 Array with platform GPL570). The lncRNA microarray analysis was performed by OE Biotech, Shanghai, China. R program was used to cluster the differentially expressed genes. Euclidean distance function was used for the two-dimensional unsupervised hierarchical clustering. The analysis of the expression difference between glioma and normal tissues was performed by limma package with Benjamini and Hochberg False Discovery Rate method.

### Cell line culture

Cell lines were acquired from BeNa Culture Collection (Beijing, China), including glioma cell lines (U87, U251, SHG-44 and U-118MG), human astroglia cell line (HA), and human embryonic kidney cell line (HEK-293). Cells were maintained in high-glucose Dulbecco’s Modified Eagle medium (DMEM, Invitrogen, Carlsbad, CA, USA) with 10% fetal bovine serum (FBS, Invitrogen, Carlsbad, CA, USA) in 5% CO_2_ at 37 °C.

### QRT-PCR

Total RNA extraction was performed using TRIzol reagent (Invitrogen). 200 ng of RNA quantified by NanoDrop2000 (Thermo Fisher Scientific, Waltham, MA, USA) was reverse transcribed using ReverTra Ace qPCR RT Kit (Toyobo, Japan) and then amplified with target gene-specific primers by the THUNDERBIRD SYBR qPCR Mix (Toyobo). Sequences of the primers (all primers were designed and synthesized by Shanghai GenePharma company) are listed in Table 1. The reaction process for PCR was initial denaturation (94 °C, 2 min), denaturation (94 °C, 30 s), primer annealing (54 °C, 30 s), primer extension (72 °C, 1 min), 30 cycles and further extension (72 °C, 10 min). The expression levels of NEAT1, *SOX2* and miR-132 were calculated by 2^− △  △ *CT*^ method. NEAT1 and *SOX2* expression levels were normalized to GADPH while miR-132 level was normalized to U6. Each experiment was performed in triplicate and each measure was done in triplicate too.

**Table 1:** Primers for qRT-PCR

Gene	Sequences
NEAT1 forward	5’-TGGCTAGCTCAGGGCTTCAG-3’
NEAT1 reverse	5’-TCTCCTTGCCAAGCTTCCTTC-3’
SOX2 forward	5’-ATTTATTCAGTTCCCAGTCCAAGC-3’
SOX2 reverse	5’-CCCTCTCCCCCCACGC-3’
miR-132 forward	5’-TGGATCCCCCCCAGTCCCCGTCCCTCAG-3’
miR-132 reverse	5’-TGAATTCGGATACCTTGGCCGGGAGGAC-3’
GAPDH forward	5’-TGAACGGGAAGCTCACTGG-3’
GAPDH reverse	5’-TCCACCACCCTGTTGCTGTA-3’
U6 forward	5’-CTCGCTTCGGCAGCACA-3’
U6 reverse	5’-AACGCTTCACGAATTTGCGT-3’

Forward: Forward primer; Reverse: Reverse primer

### Western blot analysis

Whole cell lysate of cells was employed to extract total proteins using RIPA buffer (Beyotime, Shanghai, China). Briefly, RIPA buffer was thawed and mixed well. Before using, PMSF was added to RIPA buffer and reached a final concentration of 1 mM. Cells were washed using PBS. -150 μl RIPA buffer was added to every well of the plate. After centrifugation (10,000 g, 5 min), the supernatant was collected for western blot assay. 100 μg of total protein was quantified by Pierce BCA Protein Assay Kit (Pierce, Rockford, IL, USA) and electrophoretically transferred to a PVDF membrane (Life Technologies, Gaithersburg, MD, USA). The membrane was incubated in turn with TBST supplemented 5% skimmed milk (room temperature, 1 h), primary antibody (4 °C, overnight), and secondary antibody (room temperature, 1.5 h). Anti-*SOX2* (BA3292) and anti-β-actin (BM3873) from Bosterbio (Wuhan, China) were diluted to 1:200 and used as primary antibodies. HRP-linked anti-human IgG (BM1921, 1:200, Bosterbio) was the secondary antibody. The visualization was enhanced by ECL Plus (Life Technologies, Gaithersburg, MD, USA). β-actin was the internal control. Each experiment was performed in triplicate and each measure was done in triplicate too.

### Cell transfection

Negative control (NC), miR-132 mimics, miR-132 inhibitor, si-NC, si-NEAT1, si-*SOX2*, pcDNA3.1 plasmids, pcDNA3.1-NEAT1 and pcDNA3.1-*SOX2* were all obtained from Genepharma. The cell suspension was prepared by pancreatin digested U87 and U251 cells and complete medium. Then the cells were incubated in six-well plates (1 × 10^6^ cells/well) for 18–24 h. Three hours before transfection, glioma cells at 80–90% confluence were incubated with fresh medium without serum and antibiotics. Transfection was performed using Lipofectamine 2000 (~ 0.6 μg Lipofectamine reagent/1 μg DNA, Life Technologies). Sequences of purchased siRNA, mimics and inhibitor are listed in Table 2.

**Table 2:** Purchased siRNA and mimics/inhibitor

Name	Sequences
si-NC	5’-CCCACCAGUUUGAGAC UCCACAAAU-3’
si-NEAT1	5’-GGTCTGTGTGGAAGGAGGAAGGCAG-3’
si-SOX2	5’-CUGCAGUACAACUCCAUGAUU-3’
miR-132 mimics	5’- UGUCAGUUUGUCAAAUACCCCA-3’
miR-132 inhibitor	5’- TGGGGTATTTGACAAACTGACA − 3’

‘si’ is the short name of short-interferon RNA

### Dual luciferase reported assay

HEK-293 cells were cultured in 12-well plate and co-transfected with pcDNA3.1 plasmid vectors carrying either wild or mutated NEAT1 or *SOX2* 3’UTR sequences together with miR-132 mimics or mimics NC. The NEAT1 and *SOX2* 3’ UTR constructs were purchased from GenePharma (Shanghai, China). Firefly and Renilla luciferase activities were measured 48 h after transfection using the dual-luciferase reporter assay (Promega, Madison, WI, USA). The luciferase activity was calculated as the ratio of firefly luciferase intensity/renilla luciferase intensity.

### MTT assay

Transfected glioma cells were seeded in a 96-well plate (1 × 10^4^ cells/well) to incubate for 24, 48, 72 and 96 h. Then 10 μl of 5 mg/mL MTT prepared in PBS (pH = 7.4, Sigma-Aldrich, St. Louis, MO, USA) was added to each well for incubation (4 h). After the supernatants were removed, dimethyl sulfoxide (DMSO, Thermo Fisher Scientific, Waltham, MA, USA) was added (100 μl/well) and the absorbance value (at 490 nm) was measured by a microplate reader. Each experiment was performed in triplicate and each measure was done in triplicate too.

### Transwell assay

To investigate cell migration ability, Transwell assay was conducted using a 24-well insert with 8 μm pores (Corning Incorporated, Corning, NY, USA). Cells were firstly dissociated by pancreatin, then resuspended in 100 μl serum-free medium (with 1% FBS), and finally placed in the upper chamber. In the lower chamber, 500 μl 10% FBS medium was added. The cell density was 5 × 10^5^ cells/ml. After incubation at 37 °C for 6 h, the cells on the upper side of membrane were scraped off, while those on the lower side were observed using a microscope. Before observation, the cells were fixed with 4% paraformaldehyde and stained with 0.1% crystal violet for 30 min. As for assessing cell invasion, 50 μl diluted Matrigel™ (1:8, Sigma-Aldrich, St Louis, MO, USA) was added to the upper chamber of the transwell. 500 μl 10% FBS medium was added in the lower chamber. The cell density was 5 × 10^5^ cells/ml was added in upper chamber resuspended in 100 μl 10% FBS medium. After incubation at 37 °C for 36 h, the cells on the upper side of membrane were scraped off, while those on the lower side were observed using a microscope. Each experiment was performed in triplicate and each measure was done in triplicate too.

### Statistical analyses

The form of mean ± standard errors was used to express the data. To compare the differences among two or multiple groups, paired Student’s *t* test or One-Way ANOVA were applied. *P* < 0.05 signified statistical significance. The data analysis software used was GraphPad Prism 6.0.

## Results

### LncRNA NEAT1 was up-regulated in glioma tissues and cell lines

**Fig. 1:**
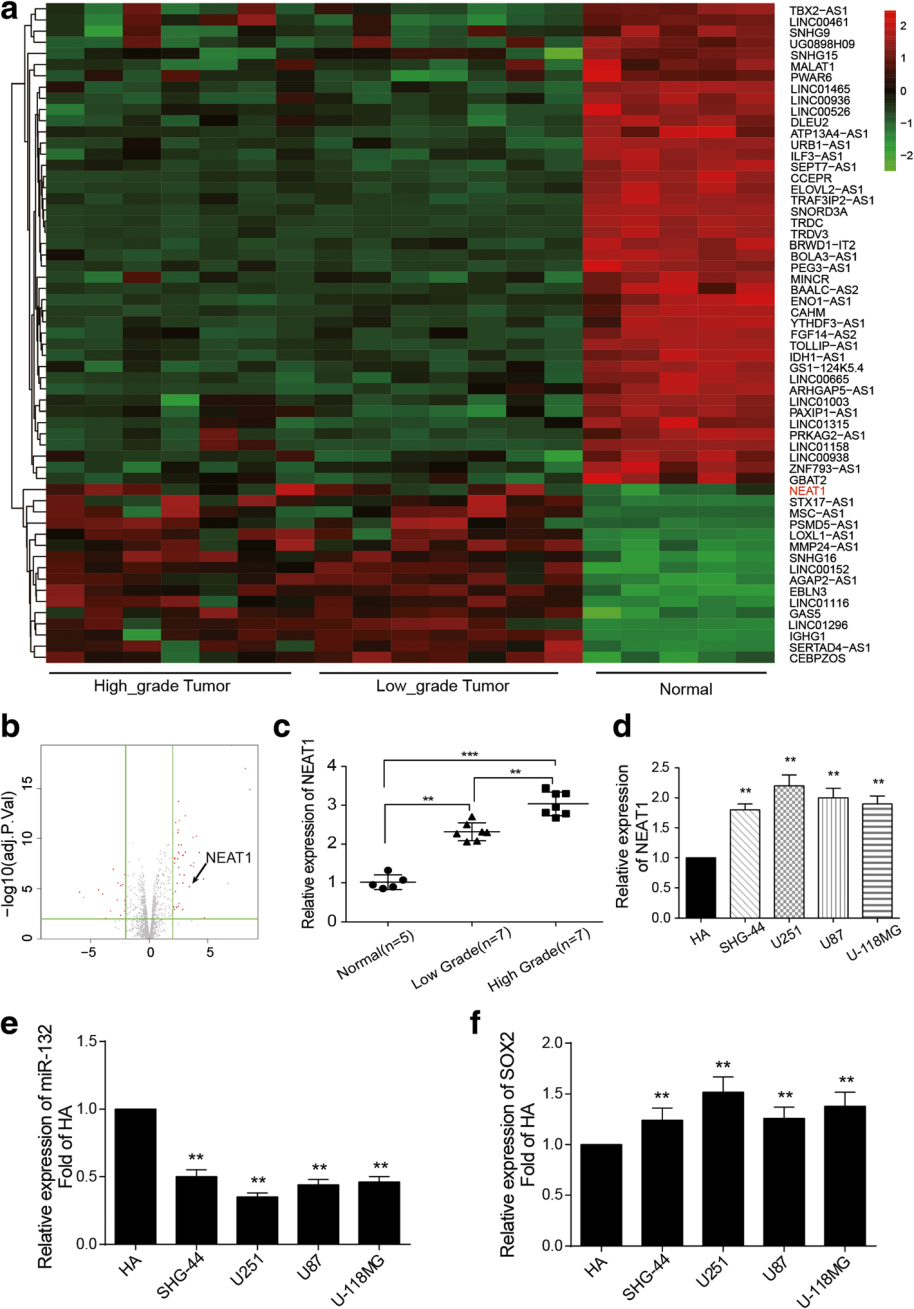
LncRNA NEAT1 is up-regulated in glioma tissues and cell lines**. a** The heat map of microarray analysis reflects the differentially expressed lncRNAs in tumor tissues. NEAT1 is one of the up-regulated lncRNAs in glioma tissue. N (high-grade tumor) = 7; N (low-grade tumor) = 7; N (normal) =5. **b** The volcano plot reflects the differentially expressed lncRNAs with fold change > 2 and *P* < 0.01. **c** The relative expression level of NEAT1 in normal tissue is lower compared with that in glioma tissue. NEAT1 expression level in low grade (stage I and stage II) glioma tissue is also lower than that in high grade (stage III and stage IV) glioma tissue. GAPDH is the internal control. N (high-grade tumor) = 7; N (low-grade tumor) = 7; N (normal) =5. ^**^ *P* < 0.01 and ^***^ *P* < 0.001. **d** The relative NEAT1 expressions of glioma cell lines (U87, U251, SHG44, U-118MG) are higher than that in normal cell line HA, especially for U251 and U87. GAPDH is the internal control. ^**^ *P* < 0.01 compared with HA. **e** The relative expressions of miR-132 in glioma cell lines (U87, U251, SHG44, U-118MG) are lower than that in normal cell line HA. ^**^ *P* < 0.01 compared with HA. (F) The relative expressions of *SOX2* in glioma cell lines (U87, U251, SHG44, U-118MG) are higher than that in normal cell line HA. ^**^ *P* < 0.01 compared with HA

Fifty-nine lncRNAs with the fold change > 2 and *P* < 0.01 were screened out as differentially expressed lncRNAs in glioma tissues (Fig. 1a-b,). Compared with normal tissues, NEAT1 expression was obviously up-regulated in tumor tissues (Fig. 1a). Meanwhile, its expression level was positively correlated with the aggravation of glioma. NEAT1 expression was also elevated in high grade glioma tissues compared with low grade ones (Fig. 1c). In four glioma cell lines (U87, U251, SHG44, U-118 MG), the expression of NEAT1 and *SOX2* was distinctively elevated compared with normal cell line HA (Fig. 1d), whereas miR-132 expression in glioma cell lines was significantly lower than that in HA cell line (Fig. 1e). U251 and U87 cells were selected as the research object in the following experiments.

### NEAT1 knockdown suppressed the viability, migration and invasion of glioma cells

**Fig. 2:**
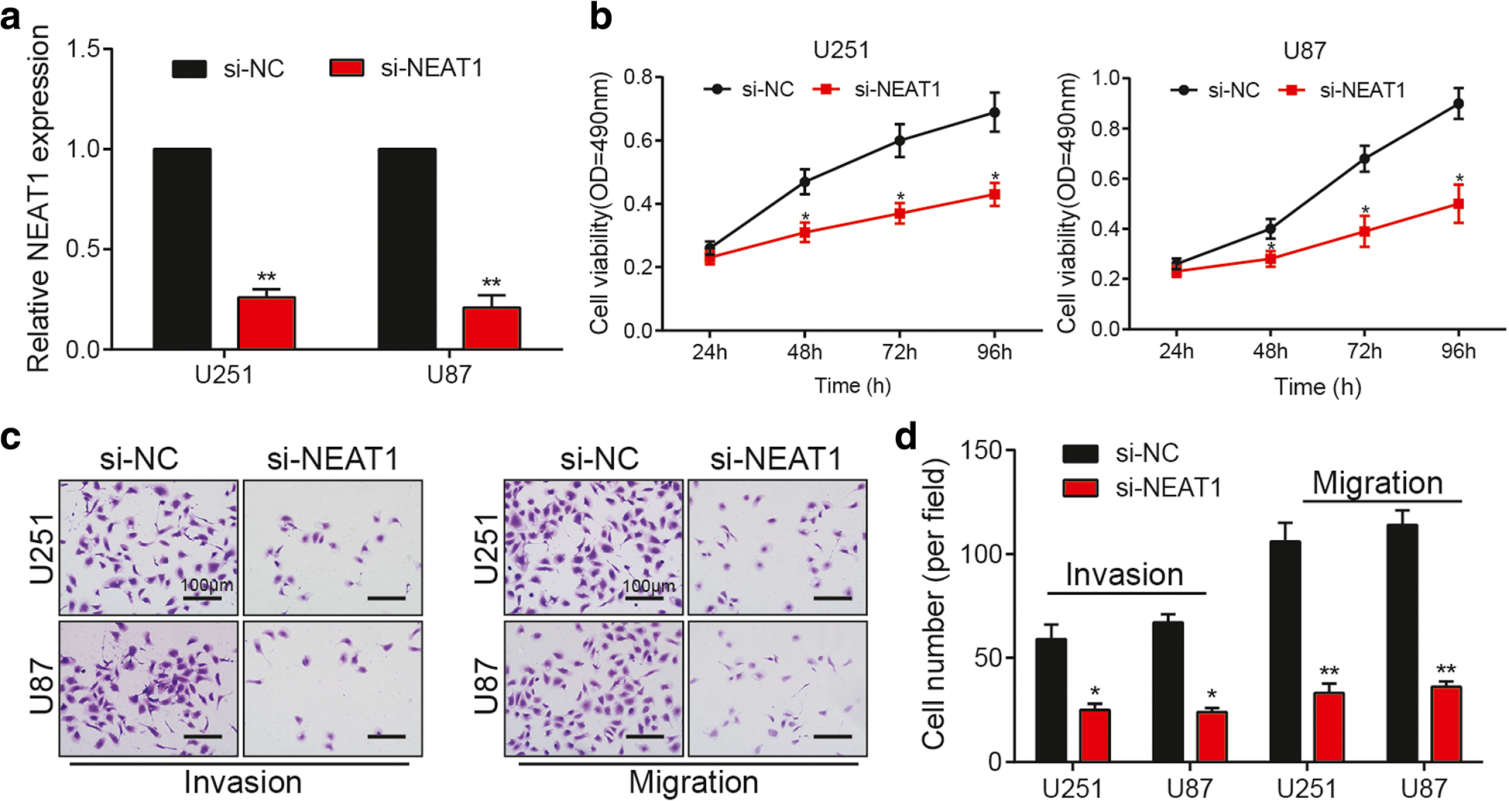
NEAT1 knockdown suppresses the viability, migration and invasion of glioma cells. **a** After being transfected with si-NEAT1, the NEAT1 expression in U251 and U87 cells is significantly reduced. GAPDH is the internal control. **b** Cell viability of U251 and U87 cells in si-NEAT1 group is significantly lower than that in si-NC group. OD: optical density. **c** The invasion and migration of U251 and U87 cells decrease in si-NEAT1 group. Scale bar: 100 μm. **d** The histogram of invasion and migration assay. ^*^ *P* < 0.05, ^**^ *P* < 0.01 compared with si-NC group (applied to all graphs). Each experiment was performed in triplicate and measured for 3 times

Figure 2a illustrated the down-regulation of NEAT1 in glioma cells after being transfected with si-NEAT1, which remarkably reduces the glioma cell viability (Fig. 2b). Meanwhile, the migratory and invasive activities of glioma cells are also obviously suppressed by NEAT1 knockdown (Fig. 2c-d). It can be concluded that NEAT1 knockdown is effective in restraining glioma cell growth and invasiveness.

### NEAT1 targeted miR-132 and miR-132 targeted *SOX2*

**Fig. 3:**
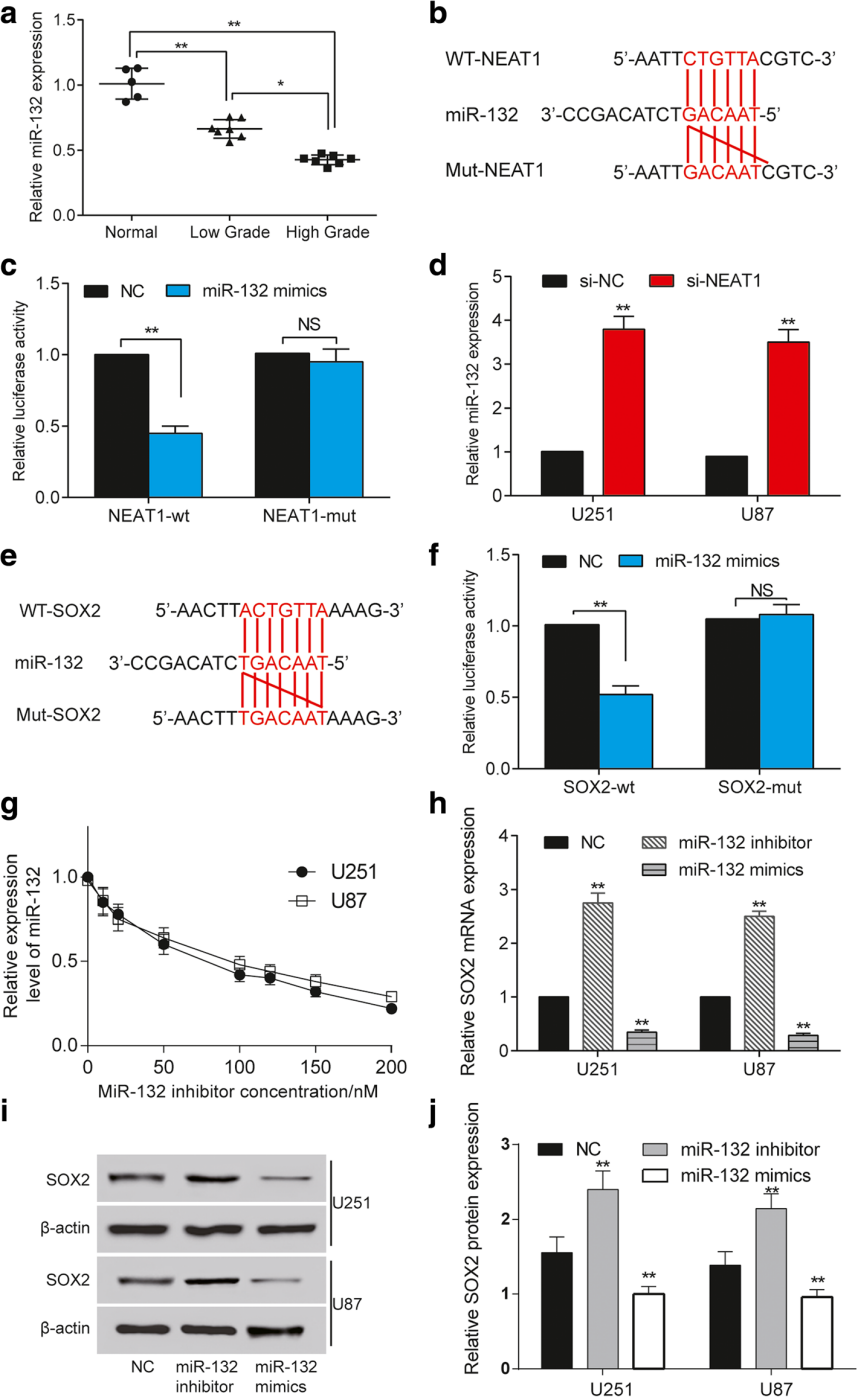
NEAT1 targets miR-132 and miR-132 targets *SOX2*. **a** The relative expression of miR-132 decrease in glioma tissue compared with that in normal tissue. The miR-132 expression is lower in high grade (stage III and stage IV) glioma tissue than that in low grade (stage I and stage II) glioma tissue. U6 is the internal control. ^*^ *P* < 0.05, ^**^ *P* < 0.01 and ^***^ *P* < 0.001. **b** MiR-132 is predicted to be a target miRNA of NEAT1. **c** The relative luciferase activity in cells co-transfected with miR-132 mimics and NEAT1-wt decrease while that in cells co-transfected with miR-132 mimics and NEAT1-mut has no significant change. ^**^ *P* < 0.01. NS: not significant. **d** The relative expression of miR-132 in glioma cells significantly increases in si-NEAT1 group. U6 is the internal control. ^**^ *P* < 0.01 compared with si-NC group. **e** *SOX2* is predicted to be a target gene of miR-132. **f** The relative luciferase activity in cells co-transfected with miR-132 mimics and *SOX2*-wt decreases while that in cells co-transfected with miR-132 mimics and *SOX2*-mut has no significant change. ^**^ *P* < 0.01. NS: not significant. **g** The dose-response diagram shows that the miR-132 inhibitor significantly suppresses miR-132 activity at concentration of 100 nM, 120 nM, 150 nM and 200 nM. We chose 120 nM in this study. **h** The relative mRNA expression of *SOX2* in glioma cells increase in miR-132 inhibitor group and decrease in miR-132 mimics group. GAPDH is the internal control. ^**^ *P* < 0.01 compared with NC group. **i** The protein expression of *SOX2* in glioma cells increase in miR-132 inhibitor group and decrease in miR-132 mimics group. β-actin is the internal control. Each experiment was performed in triplicate and measured for 3 times. **j** The histogram shows the expression of SOX2 protein in U251 and U87 cell lines. ^**^ *P* < 0.01 compared with NC group

The relative expression of miR-132 was confirmed to be down-regulated in glioma tissues, especially in high grade tumor tissues (Fig. 3a). Then we searched miRcode database to acquire the predicted potential target miRNAs of NEAT1, and we found that NEAT1 could predict to be a target of miR-132 and the relationship between NEAT1 and miR-132 have not been functionally validated (Fig. 3b). HEK293 cells co-transfected with miR-132 mimics and NEAT1-wt exhibited significant reduction in luciferase activity, confirming the target relationship between NEAT1 and miR-132 (Fig. 3c). On the contrary, the co-transfection of miR-132 and NEAT1-mut had little influence on the luciferase activity (Fig. 3c), proving that miR-132 was the target miRNA of NEAT1. After knocking down NEAT1 in glioma cells U251 and U87, miR-132 expression tremendously increased (Fig. 3d), further verifying the regulatory relationships between NEAT1 and miR-132. Similarly, through miRcode, we predicted *SOX2* as a target gene of miR-132 (Fig. 3e). The luciferase activity reflected the interaction between miR-132 mimics and wt *SOX2* 3’UTR (Fig. 3f). Before the following experiments, we confirmed the inhibition efficiency of miR-132 inhibitor. The results show that the concentrations of 100, 120, 150 and 200 nM significantly suppressed miR-132 expression (Fig. 3g). Meanwhile, *SOX2* could be up-regulated by miR-132 inhibitor and down-regulated by miR-132 mimics in glioma cells (Fig. 3h-j), demonstrating the negative regulatory effect of miR-132 on *SOX2* expression.

### *SOX2* knockdown inhibited viability, migration and invasion of glioma cell lines

**Fig. 4:**
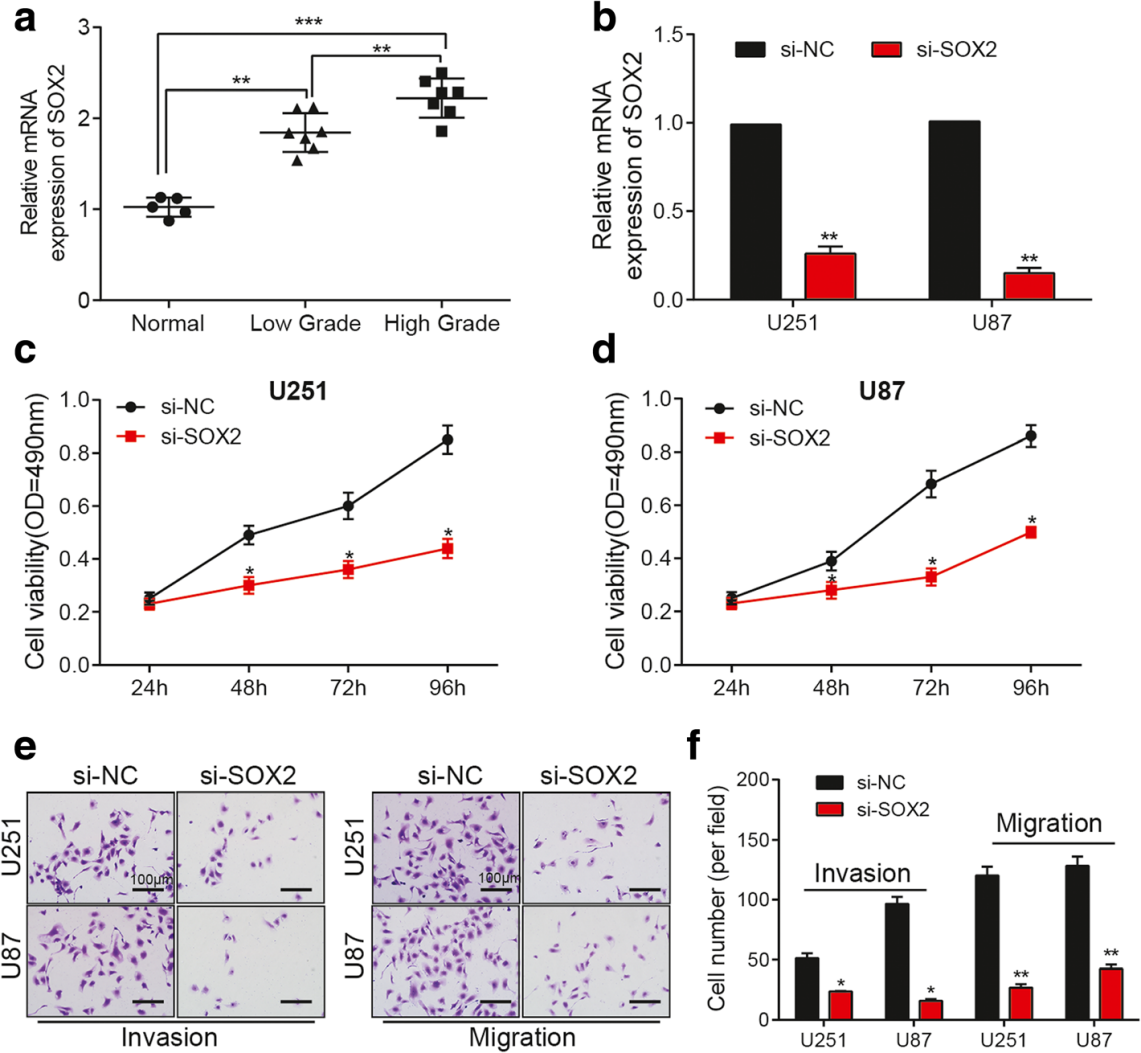
*SOX2* knockdown inhibits the viability, migration and invasion of glioma cell lines. **a** The relative expression of *SOX2* increases in glioma tissues compared with that in normal tissues. The *SOX2* expression is higher in high grade (stage III and stage IV) glioma tissues than that in low grade (stage I and stage II) glioma tissues. GAPDH is the internal control. ^**^ *P* < 0.01 and ^***^ *P* < 0.001. **b** The relative expression of *SOX2* decreases in U251 and U 87 cells in si-*SOX2* group. GAPDH is the internal control. ^**^ *P* < 0.01 compared with si-NC group. **c-d** The cell viability of U251 and U87 cells decreases in si-*SOX2* group. OD: optical density. ^*^ *P* < 0.05 compared with si-NC group. **e** The invasion and migration of U251 and U87 cells decrease in si-*SOX2* group. Scale bar: 100 μm. **f** The histogram of invasion and migration assay. ^*^ *P* < 0.05, ^**^ *P* < 0.01 compared with si-NC group. Each experiment was performed in triplicate and measured for 3 times

*SOX2* expression was up-regulated in glioma tissues, and the elevated level was positively related to the progression of glioma (Fig. 4a). In U251 and U87 glioma cells, si-*SOX2* transfection significantly reduced the expression level of *SOX2* (Fig. 4b). After knocking down *SOX2*, the viability (Fig. 4c-d) and aggressiveness (Fig. 4e-f) of glioma cells significantly decreased. Down-regulation of *SOX2* could effectively suppress the development of glioma.

### NEAT1 elevated *SOX2* expression through targeting miR-132 to promote glioma

**Fig. 5:**
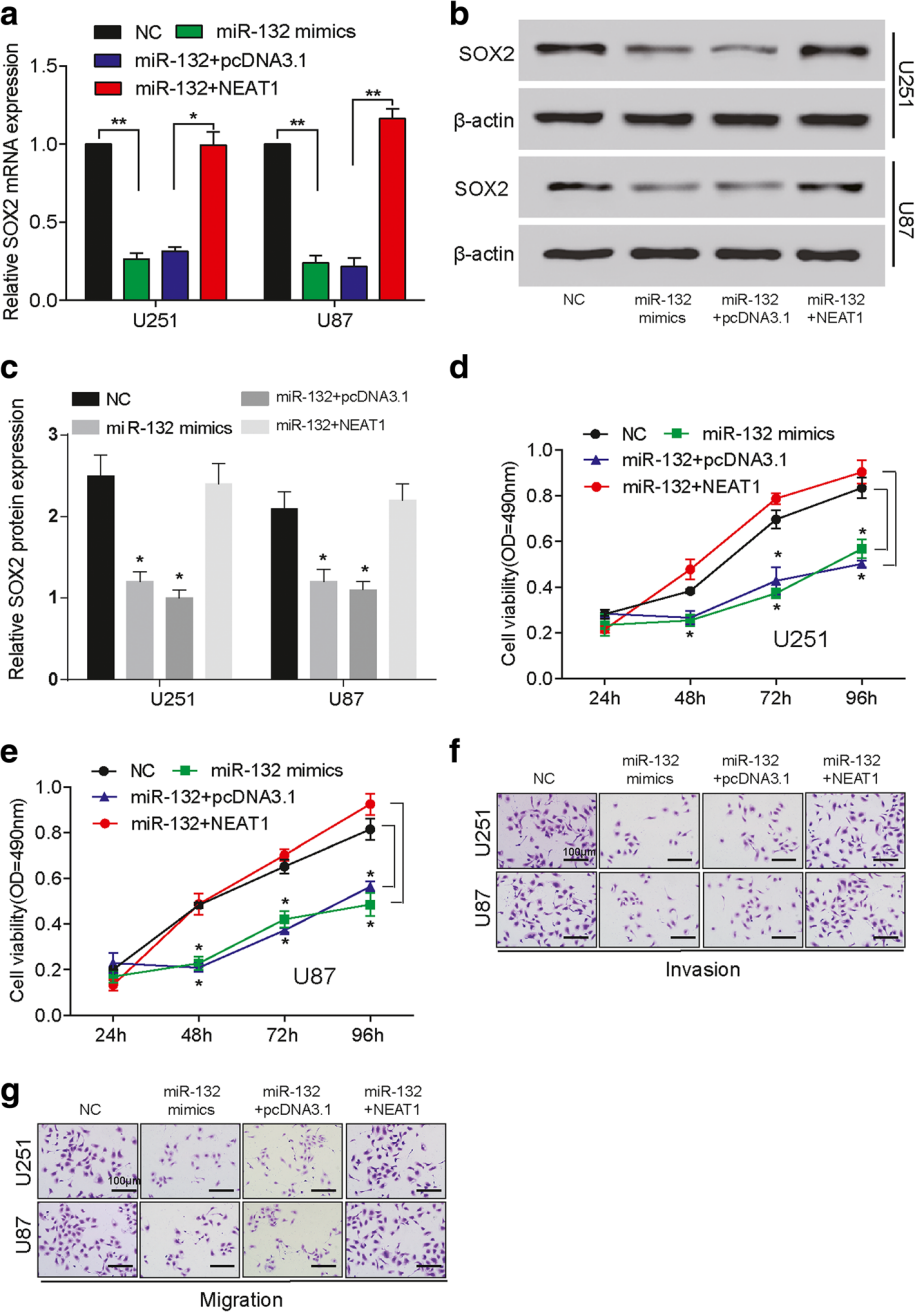
NEAT1 elevates *SOX2* expression through targeting miR-132 to promote glioma progression. **a** The relative mRNA expression of *SOX2* in U251 and U87 cells decreases in miR-132 mimics group compared with NC, increases in miR-132 + NEAT1 group compared with miR-132 + pcDNA3.1, and almost remains the same between NC and miR-132 + NEAT1 groups. GAPDH is the internal control. ^*^ *P* < 0.05 and ^**^ *P* < 0.01. **b** The protein expression of *SOX2* in U251 and U87 cells decreases in miR-132 mimics group compared with NC, increases in miR-132 + NEAT1 group compared with miR-132 + pcDNA3.1, and almost remains the same between NC and miR-132 + NEAT1 groups. β-actin is the internal control. **c** The histogram of the western blot results. **d-e** The cell viability of U251 and U87 cells in miR-132 group is similar with that in miR-132 + pcDNA3.1 group, while that in miR-132 + NEAT1 group is similar with NC group. OD: optical density. ^*^ *P* < 0.05. **f-g** The invasion and migration of U251 and U87 cells decrease in miR-132 mimics group compared with NC, and remains similar with NC in miR-132 + NEAT1 group. Scale bar: 100 μm. Each experiment was performed in triplicate and measured for 3 times

The transfection of miR-132 mimics reduced the *SOX2* expression, while the simultaneous up-regulation of miR-132 and NEAT1 returned *SOX2* expression to normal level at both mRNA and protein levels (Figure 5a-c). Glioma cell viability decreased by the transfection miR-132 mimics, and reversed by NEAT1 overexpression (Figure 5d-e). The invasive and migratory abilities of glioma cells were weakened by the up-regulation of miR-132, and offset by elevating NEAT1 expression (Figure 5f-g). Therefore, through targeting miR-132, NEAT1 could indirectly regulate *SOX2*’s expression, thus affecting glioma progression.

## Discussion

As an oncogene, lncRNA NEAT1 played a role in glioma by targeting miR-132 and then indirectly promoting *SOX2* expression. NEAT1 and *SOX2* knockdown could restrain the viability and invasiveness of glioma cells. To sum up, NEAT1 knockdown inhibited the aggression of glioma through down-regulating *SOX2* by targeting miR-132.

The effects of NEAT1 in human cancers have been thoroughly studied. For instance, NEAT1 was considered to be a competing endogenous RNA to modulate the expression of *ATAD2* by suppressing miR-106b-5p in papillary thyroid cancer [16]. NEAT1 could also be activated by *Oct4*’s binding to its promoter, which therefore led to lung cancer progression and poor prognosis outcome [17]. In addition, NEAT1 could enhance the radio-resistance of cervical cancer by suppressing miR-193b-3p-CCND1 interaction [17]. So, generally, NEAT1 could regulate cancer progression by interrupt the crosstalk between miRNA and miRNA’s target mRNA. In glioma, NEAT1 expression was elevated and positively associated with the grade of glioma. The over-expression of NEAT1 in glioma was also identified by other researchers. He et al. found that higher NEAT1 expression was closely related to higher WHO grade of glioma, which was in line with our results [18]. The up-regulation of NEAT1 in glioma stem cells was also reported by Gong et al. [8]. The aberrant expression of NEAT1 might be a biomarker of the occurrence and exacerbating of glioma.

With a high expression in tumor tissues and cells, NEAT1 exerted tumorigenesis-promotive function in glioma. The knockdown of NEAT1 could inhibit glioma cell growth and metastasis. Accumulated evidences supported our findings. For instance, it was revealed that NEAT1 down-regulation could suppress the development of glioma in vitro through restraining the mobility and viability of glioma cells as well as inducing apoptosis, which was similar to our findings [9, 19]. According to Yang et al.’s study, silencing NEAT1 reduced the proliferation but promoted the apoptosis of glioma cells CD133 and U87 [20]. Our results also confirmed the inhibitory effect of NEAT1 knockdown on the viability and invasion of glioma cells. In consequence, NEAT1 knockdown was effective in reducing aggression and proliferation of glioma cells.

In addition to NEAT1, *SOX2* was also over-expressed in glioma as well as other human tumors. *SOX2* has been identified as an oncogene that promotes squamous cancer development by promoting cancer cell viability, proliferation, migration and invasion *etc* [21–28]. Silencing *SOX2* in glioma cells had similar inhibitory effects as NEAT1 knockdown. The tumor promotive effect of *SOX2* in glioma was reported in many previous studies. Berezovsky et al. demonstrated the up-regulation of *SOX2* in glioblastoma and found that it had a higher expression in glioma with higher grade, in agreement with our study that *SOX2* expression elevated with the increasing of glioma grade [29]. In addition, Dong et al. found that the restoration of *SOX2* attenuated the anti-tumor effect of miR-429 in glioblastoma [15]. Gangemi et al. also demonstrated that *SOX2* knockdown in glioblastoma tumor-initiating cells suppressed cell growth and tumorigenicity [30]. Considering the strong association between the high *SOX2* level and glioblastoma development, some researchers believed that *SOX2* can be a target for glioma therapy in future [31]. Therefore, *SOX2* positively affected cancer development in glioma.

In our study, we identified that NEAT1 could indirectly regulate the expression of *SOX2* through targeting miR-132. NEAT1 knockdown could down-regulate *SOX2* by up-regulating miR-132, thus suppressing glioma cell growth and invasiveness. MiR-132 was considered as a suppressor in various malignancies, such as lung cancer [32], colorectal cancer [33], breast cancer [34] and ovarian cancer [35]. MiR-132 was reported to inhibit hepatocellular carcinoma cell proliferation and promote cell apoptosis by suppressing Shh/Hedgehog signaling [36]. In addition, epidermal growth factor and *TALIN2* could be suppressed by miR-132, therefore inhibit prostate cancer metastasis [37]. In pancreatic cancer, miR-132 inhibited Akt signaling and suppressed pancreatic cancer cell proliferation [38]. However, studies on its effect in glioma were not too much. Some studies proved that miR-132 was an anti-oncogene that inhibited migration and invasion of glioma [39, 40]. Nevertheless, its target relationship with NEAT1 has never been revealed except for this study. The interactions between NEAT1, miR-132 and *SOX2* in glioma were also novel findings. NEAT1 was found to function as a competing endogenous RNA in other studies, which shared a similar regulatory mechanism with our study as it regulated gene expressions through binding to miRNAs. For instance, Zhang et al. found that NEAT1 could sponge miR-485 and enhance the expression of *STAT3* in hepatocellular carcinoma [7]. In glioma, NEAT1 could specifically bind to miR-449b-5p and miR-181d-5p to inhibit their transcriptions, leading to the over-expression of oncogenes *c-Met* and *SOX5*, respectively [19, 41]. Here, we uncovered that NEAT1 could modulate *SOX2* expression in glioma by sponging miR-132.

Although we identified the promotive function of NEAT1 in glioma and investigated its relationship with miR-132 and *SOX2*, the limitations should be pointed out and made up in the future. For example, the effect of miR-132 on the viability and invasion of glioma cells needed to be further investigated. The in vivo experiments were essential to verify the in vitro results. Clinical analysis could be conducted to evaluate the association between the expression of NEAT1 or *SOX2* and the prognosis outcome of glioma patients.

## Conclusions

In summary, lncRNA NEAT1 was proved to be up-regulated in glioma and targeted miR-132. As a target gene of miR-132, *SOX2* was over-expressed in glioma. NEAT1 knockdown could inhibit the growth and invasiveness of glioma cells through indirectly down-regulating *SOX2* by targeting miR-132.

## Additional file

Additional file 1: **Table S1.** The characteristics of the included patients in this study. (DOCX 17 kb)

## Abbreviations

DMEM: Dulbecco’s Modified Eagle Medium
DMSO: Dimethyl sulfoxide
FBS: Fetal bovine serum
HA: Human astroglia cell line
HEK-293: Human embryonic kidney cell line
lncRNAs: Long non-coding RNAs
miRNAs: microRNAs
NC: Negative control
NEAT1: nuclear paraspeckle assembly transcript 1
*SOX2*: Sex determining region Y-box protein 2
