# Supplementary material for: Knockdown of long non-coding RNA NEAT1 inhibits glioma cell migration and invasion via modulation of SOX2 targeted by miR-132
Source: Mol Cancer. 2018 Jul 27;17:105. doi: 10.1186/s12943-018-0849-2 (PMC6064054; doi:10.1186/s12943-018-0849-2)
Supplement: Supplementary file 1 — Table S1. The characteristics of the included patients in this study. (DOCX 17 kb) [file 12943_2018_849_MOESM1_ESM.docx]

**Additional file**

**Table S1 The characteristics of the included patients in this study.**

| Characteristics | n | % |
| --- | --- | --- |
| Age (y)^a^ | 42±12.4 |  |
| Gender (F/M) | 8/6 |  |
| T-stage |  |  |
| T1 | 2 | 14.29% |
| T2 | 2 | 14.29% |
| T3 | 8 | 57.14% |
| T4 | 2 | 14.29% |
| Extrathyroidal extension |  |  |
| No | 5 | 35.71% |
| Yes | 9 | 64.29% |
| Lymph node metastasis |  |  |
| N0 | 2 | 14.29% |
| N1a | 4 | 28.57% |
| N1b | 8 | 57.14% |
| Distant metastasis |  |  |
| M0 | 13 | 92.86% |
| M1 | 1 | 7.14% |
| TNM stage group |  |  |
| I | 8 | 57.14% |
| II | 1 | 7.14% |
| III | 2 | 14.29% |
| IV | 3 | 21.43% |

^a^ Mean ±SD; TNM, Tumor-Lymph Node-Metastasis; n, number of patients in specified category
